# Assessment of physical activity in older Belgian adults: validity and reliability of an adapted interview version of the long International Physical Activity Questionnaire (IPAQ-L)

**DOI:** 10.1186/s12889-015-1785-3

**Published:** 2015-04-28

**Authors:** Veerle Van Holle, Ilse De Bourdeaudhuij, Benedicte Deforche, Jelle Van Cauwenberg, Delfien Van Dyck

**Affiliations:** Department of Movement and Sport Sciences, Ghent University, Watersportlaan 2, B-9000 Ghent, Belgium; Department of Public Health, Ghent University, De Pintelaan 185 4 K3, B-9000 Gent, Belgium; Research Foundation Flanders, Egmontstraat 5, B-1000 Brussels, Belgium

## Abstract

**Background:**

Adequate monitoring of older adults’ physical activity (PA) is essential to develop effective health promotion programs. The present study examined criterion validity and test-retest reliability of the long International Physical Activity Questionnaire (IPAQ-L), adapted for Belgian, community-dwelling older adults (65y and older).

**Methods:**

Participants (n = 434) completed the last seven days version of IPAQ-L, modified for the Belgian population of community-dwelling older adults. This elderly-adapted version of IPAQ-L combined vigorous and moderate activities, and questions on gait speed and recreational cycling were added. Furthermore, participants wore an ActiGraph GT3X(+) accelerometer for at least five days. Criterion validity was determined by comparing self-reported weekly minutes of moderate-to-vigorous physical activity (MVPA) and weekly minutes of total PA with accelerometer data, defined by two different cut points (Freedson vs. Copeland). To examine test-retest reliability, a subsample of 29 participants completed IPAQ-L for a second time within a ten day interval.

**Results:**

IPAQ-L showed moderate criterion validity for measuring weekly minutes of MVPA and total PA (Spearman’s *ρ* range 0.33–0.40). However, plots on agreement between self-reported and accelerometer PA showed a systematic over-reporting of IPAQ-L for MVPA. In contrast, plots indicated that IPAQ-L under-estimated levels of total PA, however, this under-estimation of total PA was substantially lower than the observed over-reporting of MVPA. Test-retest reliability was moderate-to-good for work-related PA, domestic PA, MVPA and total PA (ICC range 0.52–0.81), but poorer for transportation and recreational PA (ICC 0.44 and 0.43, respectively).

**Conclusions:**

Criterion validity results suggest that IPAQ-L is more valid to measure older adults’ weekly minutes of total PA than weekly MVPA minutes. Moreover, results might imply that content validity of IPAQ-L can be improved if specific light-intensity PA items are incorporated into IPAQ-L. Test-retest reliability of IPAQ-L was moderate to good, except for weekly minutes of transportation and recreational PA, probably due to week-to-week variability of these behaviors.

**Electronic supplementary material:**

The online version of this article (doi:10.1186/s12889-015-1785-3) contains supplementary material, which is available to authorized users.

## Background

With the worldwide increase in life expectancy, the population of older adults (≥65 years) will rise substantially during the forthcoming decades [1]. As several acute and chronic health problems (e.g., frailty) are positively associated with age [2], the growing number of elderly people also implies an increase in the number of institutionalized older adults and a higher demand on the health care sector. Therefore, development and implementation of health promotion programs for this specific age group are a public health priority. Accumulating sufficient levels of physical activity (PA) has been associated with positive health outcomes at all ages [3-6]. The promotion of “active aging” may thus serve as an effective strategy to reduce negative health outcomes in older adults. Nevertheless, to make judgments on the prevalence of older adults’ PA to identify its most important determinants, adequate monitoring of PA in this age group is essential.

The fastest and most cost-effective way to measure PA in large populations is assessing it through questionnaires. The International Physical Activity Questionnaire (IPAQ; www.ipaq.ki.se), available in a short (IPAQ-S) and a long (IPAQ-L) format, serves as the most commonly used tool to collect self-reported PA and was designed to make cross-national comparisons possible. Both formats can give an indication of total PA, but their main focus lies on measuring moderate-to-vigorous PA (MVPA). Although the short IPAQ is a good questionnaire to estimate general MVPA levels, this version was designed as a population surveillance tool, which cannot retrieve domain-specific information on PA (e.g., distinguish between transport-related and recreational activities). In contrast, IPAQ-L assesses more detailed information on the PA context and is used to evaluate population PA levels. Such context-specific indices are needed to develop effective and context-specific intervention programs, as different correlates may exist for separate PA domains [7].

IPAQ-L has been shown to have fair validity and acceptable reliability in a wide range of adult populations [8-10], but research investigating its applicability to elderly populations remains scarce [11]. To our knowledge, only two studies [12,13] examined measurement properties of IPAQ-L in a population of older adults. A first study was conducted in Serbian older adults and examined test-retest reliability of the interviewer-administered IPAQ-L within a 2-week interval. Overall, reliability was moderate to very good, with the lowest intra-class coefficient found for men’s leisure-time PA (ICC = 0.54) and the highest for women’s transport-related PA (ICC = 0.91). The second study on measurement properties of IPAQ-L, which was conducted in Hong Kong urban-dwelling older adults, compared both validity (using accelerometers as criterion measure) and reliability (2-week interval) of the Chinese interviewer-administered IPAQ-L across different neighborhood types (varying on socio-economic status and walkability). The authors concluded that IPAQ-L was reliable (ICC range across neighborhoods 0.77 - 0.93) and acceptably valid, especially for measures of walking. However, Hong Kong older adults’ PA was generally high compared to that of populations from other countries, probably due to the specific geographical characteristics of Hong Kong [13]. Validity and reliability results regarding this particular Chinese population may thus not be applicable to other populations, which stresses the need to examine applicability of the IPAQ-L in other geographic regions. Moreover, IPAQ-L used in the Hong Kong study was not specifically adapted for older adults. An older-adults-specific version of IPAQ-L may be more appropriate as some behaviors such as vigorous activities are less prevalent in this age group. Cerin et al. [13] therefore also suggested to put less emphasis on items assessing vigorous PA (or even omit them).

PA questionnaires are generally validated through examining their agreement with an objective criterion measure of PA, mostly assessed through activity monitors or accelerometers [11]. These devices, mostly worn at hip bone level, capture human-body accelerations and translate them into activity counts, which in turn give an indication of someone’s activity degree. Yet, a common issue that needs to be addressed when using accelerometers is the choice of adequate cut points to categorize different intensities of PA. Tailoring these cut points for specific age groups such as older adults is very important, because the intensity of certain daily activities (e.g., walking) can differ substantially between older and younger adults [14]. To date, the choice of accelerometer cut points remains arbitrary and studies in the same research domain have used different cut points, making comparability of their results difficult. To define MVPA, the vast majority of studies in adults (18-65y) adopted cut points recommended by Freedson et al. [15] and also PA studies in older adults have used these intensity categories [16-18]. It should be noted, however, that the Freedson cut point categorization was based on results in a sample of adults with a mean age of 25 ± 4y, whose fitness levels may be significantly higher than those of adults aged ≥65y [14]. In contrast, Copeland & Esliger [19] defined an older-adults-specific cut point for moderate PA based on energy expenditure results of Canadian older adults with a mean age of 70 ± 4 years, showing a remarkably lower number of activity counts defining moderate-intensity walking in this age group (1,041 counts.min^−1^ vs. 1,952 counts.min^−1^ defined by Freedson et al. [15]). This suggests that choosing different cut points may have large consequences on the interpretation of older adults’ PA levels [20-22] and this can also have an important impact on the validity of self-reported PA measures when using accelerometers as a criterion.

The present study aimed to examine criterion validity of an adapted version of the long International Physical Activity Questionnaire (IPAQ-L, last 7 days, interviewer-based) in a population of community-dwelling Belgian older adults, by testing its agreement with objective, accelerometer-derived measures of weekly minutes of total PA and MVPA. In their review, Kowalski et al. (2012) described an average correlation coefficient of r = 0.38 between older adults’ self-reported PA and direct measures (e.g., accelerometers). Moreover, the review of Helmerhorst et al. [11] observed median Spearman correlations of r = 0.41 between older adults’ self-reported and objectively-measured PA measures. Hence, in the present study, it was hypothesized that similar validity results would be obtained, with correlation coefficients ranging between 0.35 and 0.45. Weekly minutes of total PA and MVPA were defined using the cut points of Freedson et al. [15], as well as those of Copeland & Esliger [19]. Since the Copeland & Esliger cut point for MVPA is elderly-specific, a second hypothesis concerning validity results of this study was that we hypothesized to find higher agreement between self-reported MVPA and accelerometer-derived MVPA defined by the Copeland & Esliger cut point, compared to validity results comparing self-reports with accelerometer MVPA defined by the commonly used adults-specific Freedson cut point. In addition to assessments of an instrument’s criterion validity, it is also important to examine the stability of this instrument over time and whether it is capable of measuring a variable (e.g., PA) with consistency [23]. Hence, a second aim of this study was to assess test-retest reliability of the elderly-adapted IPAQ-L questionnaire. Previous studies on test-retest reliability of PA questionnaires in older adults reported median Intraclass Coefficient estimates of 0.65 [11] and similar estimates were hypothesized to be found in this Belgian study in older adults.

## Methods

### Sample and procedures

Community-dwelling older adults (≥65y) were recruited in Ghent and its suburbs between October 2010 and September 2012. The Public Service of Ghent provided addresses of all residents aged ≥65y and a systematic random sample of 1,750 older adults, stratified by gender and age (<75y vs. ≥75y), was drawn. An informative letter was mailed to contact selected older adults, in which the purpose of the study was explained and the visit of a trained interviewer during the subsequent two weeks (between 9 AM and 5 PM) was announced. Approximately one week later, potential participants were visited at home. In case of absence at the moment of visit, two additional attempts were made on different days and different times of the day (AM vs. PM) to reduce potential selection bias. Eligibility criteria for inclusion were as follows: participants needed to understand and speak Dutch, live independently (non-institutionalized), and be able to walk 100 m without severe physical restrictions. Eventually, 1,260 older adults were found at home when the interviewer visited them, of which 508 participated in the study. Six hundred twenty-seven people refused participation and 125 were classified as “not eligible due to severe physical restrictions”, resulting in a response rate of 44.8% (508/1,135 eligible participants found at home).

During the home visit, respondents gave written consent for participation in the study and answered a face-to-face interview, targeting demographics and PA levels (IPAQ-L) in the preceding week. Furthermore, participants were instructed how to wear an Actigraph GT3X(+) accelerometer for the next consecutive seven days, during waking hours excluding contact sports, bathing or swimming activities. Approximately one week later, the interviewer re-visited participants to collect the accelerometers. To minimize inter-rater bias, all interviewers received a standardized training before initiating data collection, in order to adequately conduct the complete home visit procedure (i.e., contacting selected older adults; explaining procedures concerning the interview and accelerometer data collection; assessment of the interviewer-administered questionnaire).

In addition, during the second home visit, IPAQ-L was reassessed in a random sample (stratified on gender) of 30 participants, in order to collect data for examining test-retest reliability of the questionnaire. This subsample’s socio-demographic characteristics were similar to those of the validity sample. For the reliability study, both assessments of IPAQ-L were conducted by the same interviewer and the mean time interval between both visits was 9.6 ± 1.7 days. The study protocol was approved by the Ethics Committee of the Ghent University hospital.

### Measures

#### Socio-demographics and physical measures

Participants self-reported their age, current living situation (responses dichotomized into “having a partner” and “having no partner”), educational level (responses dichotomized into “tertiary education” and “non-tertiary education”) and former occupational status (responses categorized into “household”, “blue collar” including workman and self-employed, and “white collar” including education/teaching; employee; executive staff member and profession). To calculate BMI (kg/m^2^), height and weight were measured using a SECA 214 stadiometer (accuracy 0.1 cm) and a SECA 813 Robusta weight scale (accuracy 0.1 kg), respectively.

#### Self-reported PA: adapted IPAQ-L interview version

Self-reported PA was assessed through the long International Physical Activity Questionnaire (last seven days interview version, http://www.ipaq.ki.se). This 19-item questionnaire was originally designed for young and middle-aged adults (15-69y) and covers four activity domains: work-related PA (paid employment, as well as voluntary work), transportation PA, domestic PA, and recreational PA. Within a time frame of the last week, IPAQ items assess frequency (reported in number of days; “During the last 7 days, on how many days did you do ..*.”*) and average duration per day (reported in hours and minutes; “How much time did you usually spend on one of those days doing …”) spent in these specific PA domains. Participants were prompted to report only those activities with a minimum length of 10 consecutive minutes.

To make this measurement tool more appropriate for estimating older adults’ time spent in PA, some modifications to the original adults questionnaire were made for the present study. An overview of all adapted items can be found in the online Additional file 1. Briefly, three types of adaptations were made. Firstly, per PA domain, items on vigorous PA were combined with items on moderate PA, because in older adults, activities of vigorous intensity might be avoided due to physical health problems or restrictions, while moderate-intensity activities may also evoke similar physiological responses (e.g., substantial rises in heart rate) for this age group [24,25]. Secondly, since walking is the most prevalent type of PA in older adults [26], items on walking were completed with an item assessing gait speed (i.e., low; moderate; high pace). Low-pace walking in older adults is classified as “light PA” [27]. Adding a gait speed item to IPAQ-L enables researchers to make a distinction between light- and moderate intensity walking. Thirdly, items on recreational cycling were added. Adding recreational cycling items to IPAQ is relevant for Europeans, given the generally high cycling prevalence in Europe compared to other regions (e.g., North-America) [28,29]. Besides, since a study in 48,879 Flemish older adults showed that 53.8% of the participants reported to walk or cycle for recreation at least once a week [30], it was considered useful to add an item on recreational cycling to the elderly-adapted IPAQ.

According to the cultural adaptation guidelines provided by the IPAQ core group (https://sites.google.com/site/theipaq/cultural-adaptation), all newly generated items (see Additional file 1) were for- and backward translated from Dutch to English and vice versa by two independent professional linguists. Translations confirmed that the new items’ content had not changed during the translation process. Next, the adapted IPAQ-L questionnaire was pilot tested in a convenience sample (n = 4) of community-dwelling Flemish older adults in order to check its feasibility for this age group. After confirmation of all items’ clarity and relevance, data collection was initiated in the current sample (n = 508). An overview of adapted IPAQ-L questionnaire’s content is shown in Additional file 2.

Weekly minutes of PA were calculated for each specific reported PA behavior. Weekly minutes of total MVPA were calculated by summing weekly minutes of all reported PA behaviors, excluding walking at low pace for work-related, transportation and recreational walking, respectively. This new variable “Total MVPA” was truncated at a maximum of 1680 weekly minutes (~4 hours.day^−1^). In addition, this variable was dichotomized according to the Public Health Recommendations (150 min.week^−1^). Next, weekly minutes of time spent doing work-related PA, transportation PA, domestic PA, and recreational PA were calculated and subsequently truncated to a maximum of respectively 1800 (~6 hours.working day^−1^), 1260 (~3 hours.day^−1^), 1680 (~4 hours.day^−1^), and 1680 (~4 hours.day^−1^) weekly minutes. Truncated domain-specific variables were summed to create “Total PA”, which was truncated at a maximum of 2520 weekly minutes (~6 hours.day^−1^). Data were truncated according to the truncation rules described by Dubuy et al. [31], previously applied in Flemish adults. Data truncation is a commonly used method and is also described in the IPAQ scoring protocol guidelines (https://sites.google.com/site/theipaq/scoring-protocol). However, this IPAQ scoring protocol only provides separate data truncation rules for total walking, total moderate and total vigorous PA. In the present study, it was decided to apply domain-specific data truncation rules on the summary variables for transport, domestic, leisure-time and work-related PA, respectively [31], as our adapted version of IPAQ-L does not distinguish moderate from vigorous PA.

#### Criterion measure: accelerometer-based MVPA

Criterion validity of the last 7 days interviewer-assessed IPAQ-L in older adults for measuring MVPA was examined through testing its agreement with an objective MVPA measure, measured through Actigraph GT3X(+) accelerometers. These small (1.5”×1.44”×0.70”) and lightweight (27 g) solid-state devices can capture three-axis human-body accelerations, ranging from 0.5 to 2.5 *g*, digitized at a rate of 30 Hz, which are expressed as “counts”. These counts can then be summed over a specific period or “epoch”, defined by the user and varying across age groups. For the present study, 60s epochs were used, according to the recommendations for older adult samples [32]. Accelerometers were attached to an adjustable elastic waist belt and worn above the right hip bone for at least five, to preferably seven consecutive days. In this study, only data capturing the vertical plane were used to estimate participants’ time spent in MVPA.

Raw accelerometer data were downloaded with the Actilife 6.0 software (Actigraph, Fort Walton Beach, FL, USA) and subsequently screened, cleaned and scored using MeterPlus 4.3 (Santech, Inc.; www.meterplussoftware.com). A valid day was defined as a minimum of 10 wearing hours and only participants with accelerometer data on at least five valid days were included for analysis (25 participants were excluded based on this criterion). Periods covering ≥ 90 minutes of consecutive zeros were defined as “non-wearing”, as recommended by Choi et al. [33]. Two separate cut points defining MVPA were applied. For the Freedson [15] cut point, MVPA was defined as ≥ 1,952 counts.min^−1^, whereas the Copeland & Esliger [19] cut point defined MVPA as ≥ 1,041 counts.min^−1^. Both accelerometer MVPA outcomes will be respectively referred to as “Freedson MVPA” and “Copeland MVPA”. In addition, both accelerometer-derived MVPA variables were dichotomized according to the Public Health Recommendations for PA (all MVPA records <150 min.wk^−1^ = 0; all MVPA records ≥150 min.wk^−1^ = 1).

#### Criterion measure: accelerometer-based total PA

Similar to the difference in Freedson MVPA and Copeland MVPA cut points, sedentary behaviors are also defined by different cut points according to Freedson et al. [15] and according to Copeland & Esliger [19]. Depending on whether Freedson vs. Copeland & Esliger cut points were used, sedentary behaviors were defined as all accelerometer data of ≤ 100 counts.min^−1^ or ≤ 50 counts.min^−1^, respectively. Total accelerometer PA was calculated as the sum of all counts.min^−1^ above these thresholds and will be referred to as “Freedson total PA” (>100 counts.min^−1^) and “Copeland total PA” (>50 counts.min^−1^) in the further sections of this paper.

### Statistical analyses

**Figure 1:**
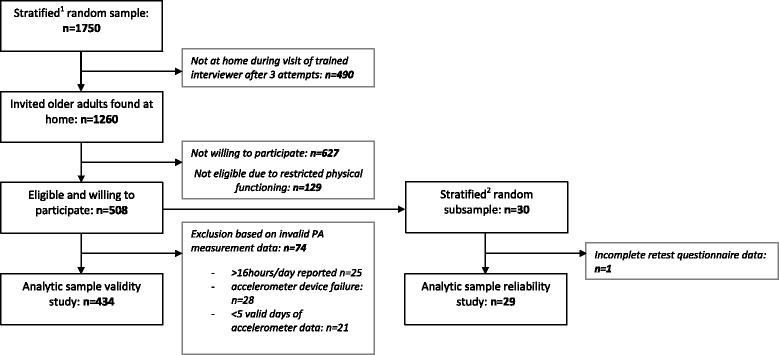
Flowchart of the recruitment procedures. Legend: ^1^stratified on gender and age (<75y vs. ≥75y); ^2^stratified on gender.

Analyses were conducted in SPSS 19.0 (Chicago, IL, USA) and statistical significance was set at p < 0.05. Based on the data, 74 participants of the validity study were excluded before analyses were conducted (see Figure 1). Twenty-five participants were excluded because their total reported levels of PA exceeded 6720 weekly minutes (~16 hours/day) [34]. Twenty-eight participants were excluded because of accelerometer failure and 21 participants had fewer than five valid days of accelerometer data. The total analytic sample for the validity study consisted of 434 participants.

Summary data for total PA and MVPA were compared between measurement methods (i.e., self-reported vs. Freedson vs. Copeland) using Repeated Measures ANOVA tests. Validity of self-reported weekly minutes of MVPA and total PA were determined through calculating correlation coefficients between respectively self-reported weekly minutes of MVPA and total PA and accelerometer-derived weekly minutes of MVPA and total PA. Nonparametric Spearman rank order correlation coefficients were calculated because the data were non-normally distributed [35]. Correlation coefficients ≥0.40 were considered as good validity, coefficients between 0.30 and 0.40 were classified as moderate, and coefficients <0.30 were categorized as poor validity [23]. Moreover, differences between self-reported and accelerometer-derived PA (MVPA and total PA) were plotted against the average of both, in order to create Bland-Altman plots for illustrating systematic error and limits of agreement (LOA) [36,37]. As for both MVPA and total PA plots, differences were significantly related to the averages, a linear regression approach was proposed, and limits of agreement could be calculated by regressing the absolute values of the residuals on the averages [37]. However, residuals were not normally distributed, which is a requirement for creating parametric Bland-Altman plots [36]. Therefore, as recommended by Bland and Altman [37], a nonparametric approach had to be applied in the present study. According to the non-parametric method, the difference between self-reported PA (using IPAQ-L) and accelerometer-derived PA was calculated and expressed as a percentage of difference (self-report data as a percentage of accelerometer data). This variable was plotted against the average of accelerometer-derived PA, which was subdivided into quartiles in the present study. In total, sixteen plots were created (one for each quartile of Freedson MVPA, Copeland MVPA, Freedson total PA, and Copeland total PA, respectively). The nonparametric LOA equivalents were obtained by calculating the values outside which 10% of the observations fell. Specifically, for each of the 16 above-mentioned plots, 5th and 95th percentiles of the percentage-of-difference measures were calculated and subsequently superimposed on the scatter diagram, reflecting 90% limits of agreement. 95% confidence intervals around these limits were calculated by bootstrapping methods, using the standard errors of these 5th and 95th percentiles [37].

To examine test-retest reliability of the interviewer-assessed IPAQ-L in older adults, two reliability calculations were applied. Single measures Intra-class correlation coefficients (ICC’s; two-way mixed methods, absolute agreement) with 95% confidence intervals were calculated, comparing participants’ test-retest self-reported weekly minutes spent in four PA domains (work-related PA, transportation PA, domestic PA, and recreational PA) and summary measures of self-reported MVPA and total PA. In the present study, ICC estimates >0.75 were considered as good reliability scores, ICC’s between 0.50 and 0.75 were classified as moderate, and ICC’s <0.50 indicated poor reliability [23]. The total analytic sample of the reliability analysis consisted of 29 participants, as one participant was excluded based on incomplete retest questionnaire data (see Figure 1).

## Results

### Sample characteristics and PA levels

Sample characteristics and PA levels (weekly minutes) of the validity (n = 434) and the reliability subsample (n = 29) are presented in Table 1. Mean age, BMI, gender distribution, proportion of former white collar workers and proportion of older adults living with a partner were similar across both samples. In contrast, the samples differed in educational level, with more highly educated people being represented in the reliability subsample. For the total sample, participants’ mean age was 74 ± 6 years, mean Body Mass Index was 27 ± 4 kg/m^2^ (32% normal weight, 43% overweight, 25% obese), and 66% lived with a partner. Fifty-four percent were women, which is similar to the gender distribution in Belgium (54% women; [38]). In contrast, compared to the Belgian population of older adults, a higher percentage of participants lived with a partner (65.8% versus 56.2% for Belgium; [38]).

**Table 1:** **Sample characteristics and physical activity by measurement method**

**Socio-demographics**	**Total sample (n = 434)**		**Subsample (n = 29)**	
	**Mean ± SD**		**Mean ± SD**	
Gender (% female)	53.7		51.7	
Age in years	74.2 ± 6.2		76.6 ± 6.4	
Body Mass Index in kg/m^2^	27.4 ± 4.4		27.2 ± 3.8	
Living situation (% having a partner)	66.2		69.0	
Educational level (% tertiary)	38.5		50.0	
Main former occupation				
White collar (%)	55.0		62.1	
Blue collar (%)	27.1		20.7	
Household (%)	17.9		17.2	
**Physical activity**	**Mean ± SD**	**Median (IQR)**	**Mean ± SD**	**Median (IQR)**
Work-related PA^1^ in min.week^−1^	31.8 ± 125.7	0.0 (0.0-0.0)	13.1 ± 70.6	0.0 (0.0-0.0)
Transportation PA^1^ in min.week^−1^	123.4 ± 162.5	60.0 (0.0-80.0)	203.5 ± 168.1	140.0 (90.0-305.0)
Domestic PA^1^ in min.week^−1^	361.3 ± 373.2	240.0 (43.8-600.0)	329.1 ± 307.1	255.0 (45.0-545.0)
Recreational PA^1^ in min.week^−1^	171.2 ± 245.8	68.0 (0.0-240.0)	109.5 ± 172.2	45.0 (0.0-140.0)
MVPA in min.week^−1^				
Self-reported MVPA^a^	630.1 ± 492.8	540.0 (210.0-948.8)	638.1 ± 580.0	580.0 (192.5-977.5)
Freedson MVPA^b^	111.5 ± 116.8	71.5 (23.7-162.9)	118.6 ± 78.3	120.2 (49.0-196.0)
Copeland MVPA^c^	326.5 ± 240.9	283.5 (136.0-461.8)	339.4 ± 363.0	363.0 (217.0-465.0)
PHR 150 min. week^−1^ (% achieving)				
Self-reported MVPA^a^	81.6		79.3	
Freedson MVPA^b^	27.4		33.3	
Copeland MVPA^c^	71.2		81.5	
Total PA in min.week^−1^				
Self-reported^a^	687.3 ± 577.5	577.5 (268.8-992.5)	655.1 ± 580.0	580.0(257.5-977.5)
Freedson cut point^d^	1911.3 ± 631.9	1932.0 (1459.9-2341.9)	1898.4 ± 661.5	1934.0(1538.8-2283.0)
Copeland & Esliger cut point^e^	2304.4 ± 658.8	2308.2 (1826.3-2761.5)	2272.3 ± 688.8	2236.0(1800.0-2604.0)

^a^Self-reported PA measured during the second visit.

^b^Accelerometer-derived MVPA ≥ 1952 counts.min^−1^.

^c^Accelerometer-derived MVPA ≥ 1041 counts.min^−1^.

^d^Accelerometer-derived total PA > 100 counts.min^-1^.

^e^Accelerometer-derived total PA > 50 counts.min^-1^.

PHR = Public Health Recommendations; PA = physical activity; MVPA = moderate-to-vigorous physical activity; IQR = interquartile range.

Table 1 further shows that for the total sample, the highest levels of weekly minutes of total PA were found for Copeland total PA (median = 2308.2 min.week^−1^, equivalent to 5.5 hours.day^−1^), whereas the lowest number of total PA minutes were self-reported (median = 577.5 min.week^−1^, or 1.4 hours.day^−1^; p < 0.001). However, for weekly minutes of MVPA, self-reported activity levels were highest (median = 540.0 min.week^−1^, or 1.3 hours.day^−1^), followed by Copeland MVPA (median = 283.5 min.week^−1^, or 0.7 hours.day^−1^), and Freedson MVPA (median = 111.5 min.week^−1^, or 0.3 hours.day^−1^), respectively (p < 0.001). Moreover, a substantially smaller proportion of participants achieved the Public Health Recommendation for MVPA (≥150 min.wk^−1^) when it was defined by the Freedson cut point (27.4%), compared to Copeland MVPA (71.2%), and self-reported MVPA (81.6%). Regarding domain-specific PA, participants reported to have spent most of their time doing domestic PA (median = 240.0 min.week^−1^, or 0.6 hours.day^−1^). Less time was spent doing recreational PA (median = 68.0 min.week^−1^, or 0.2 hours.day^−1^), transportation PA (median = 60.0 min.week^−1^, or 0.1 hours.day^−1^) and work-related activities (median = 0.0 min.week^−1^).

### Criterion validity

#### Validity for measuring weekly minutes of MVPA

**Figure 2:**
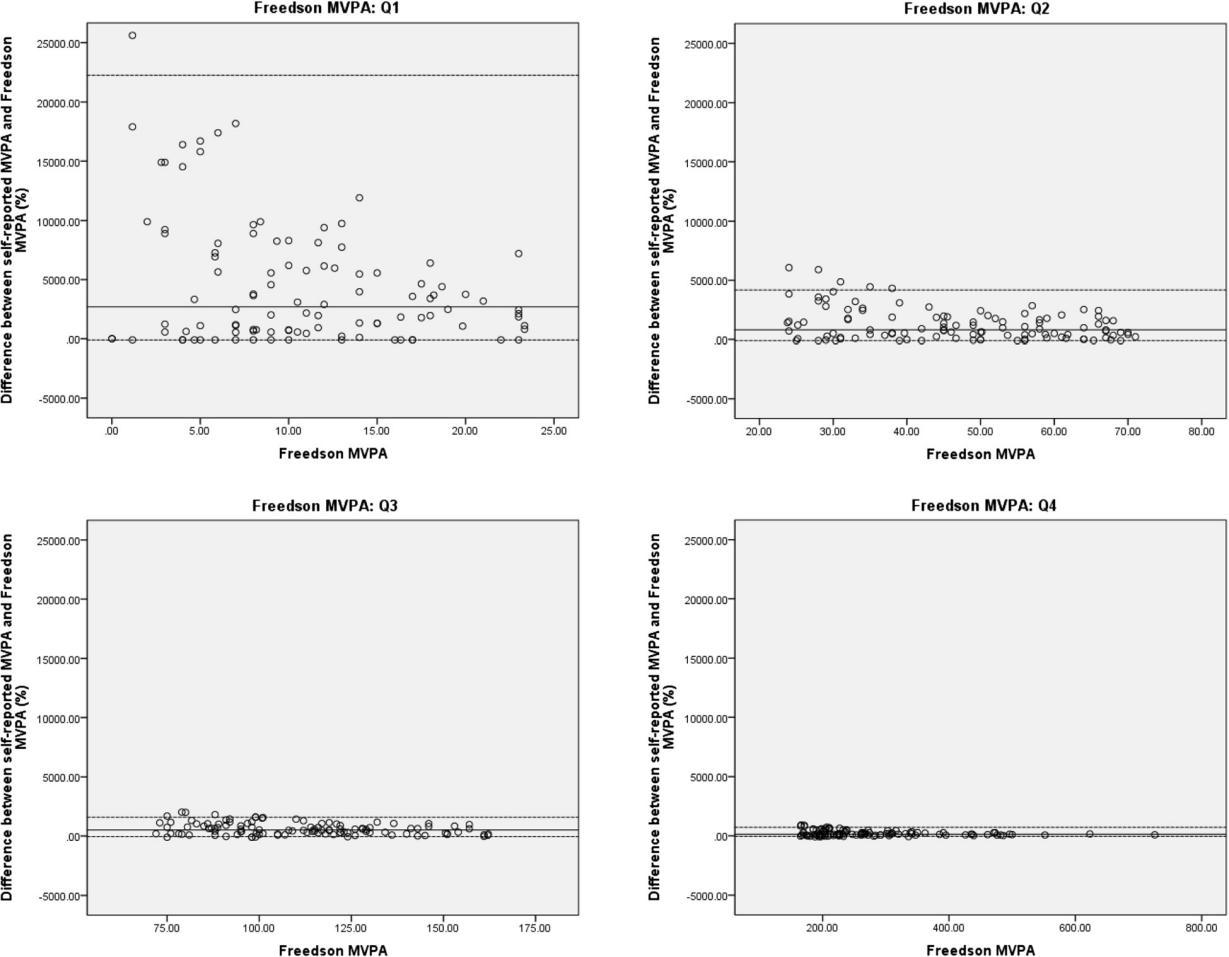
Difference between self-reported MVPA and Freedson MVPA for each quartile of Freedson MVPA. Legend: MVPA = moderate-to-vigorous physical activity; Freedson MVPA = weekly minutes of accelerometer-derived moderate-to-vigorous physical activity ≥1952 counts.min^−1^; Q1 = first quartile; Q2 = second quartile; Q3 = third quartile; Q4 = fourth quartile. y-axis represent differences between self-reported MVPA and Freedson MVPA, expressed as a percentage; x-axis represent quartiles of Freedson MVPA. Full lines represent median (M) percentage of difference, dotted lines show the 90% nonparametric limits of agreement (LOA), representing 5th and 95th percentiles (P5 and P95): **Q1**: M = 2689.5; P5 = −99.9 (95% CI for P5: −99.9 - -99.8); P95 = 22271.3 (95% CI for P95: 15800.0 – 39200.0); **Q2**: M = 799.9; P5 = −99.9 (95% CI for P5: −99.9 - -48.3); P95 = 4177.2 (95% CI for P95: 3203.7 – 5383.9); **Q3**: M = 515.0; P5 = −46.9 (95% CI for P5: −95.0 – 55.7); P95 = 1597.0 (95% CI for P95: 1360.1 – 1904.5); **Q4**: M = 128.8; P5 = −45.4 (95% CI for P5: −75.2 - -13.3); P95 = 708.54 (95% CI for P95: 585.7 – 853.5).

**Figure 3:**
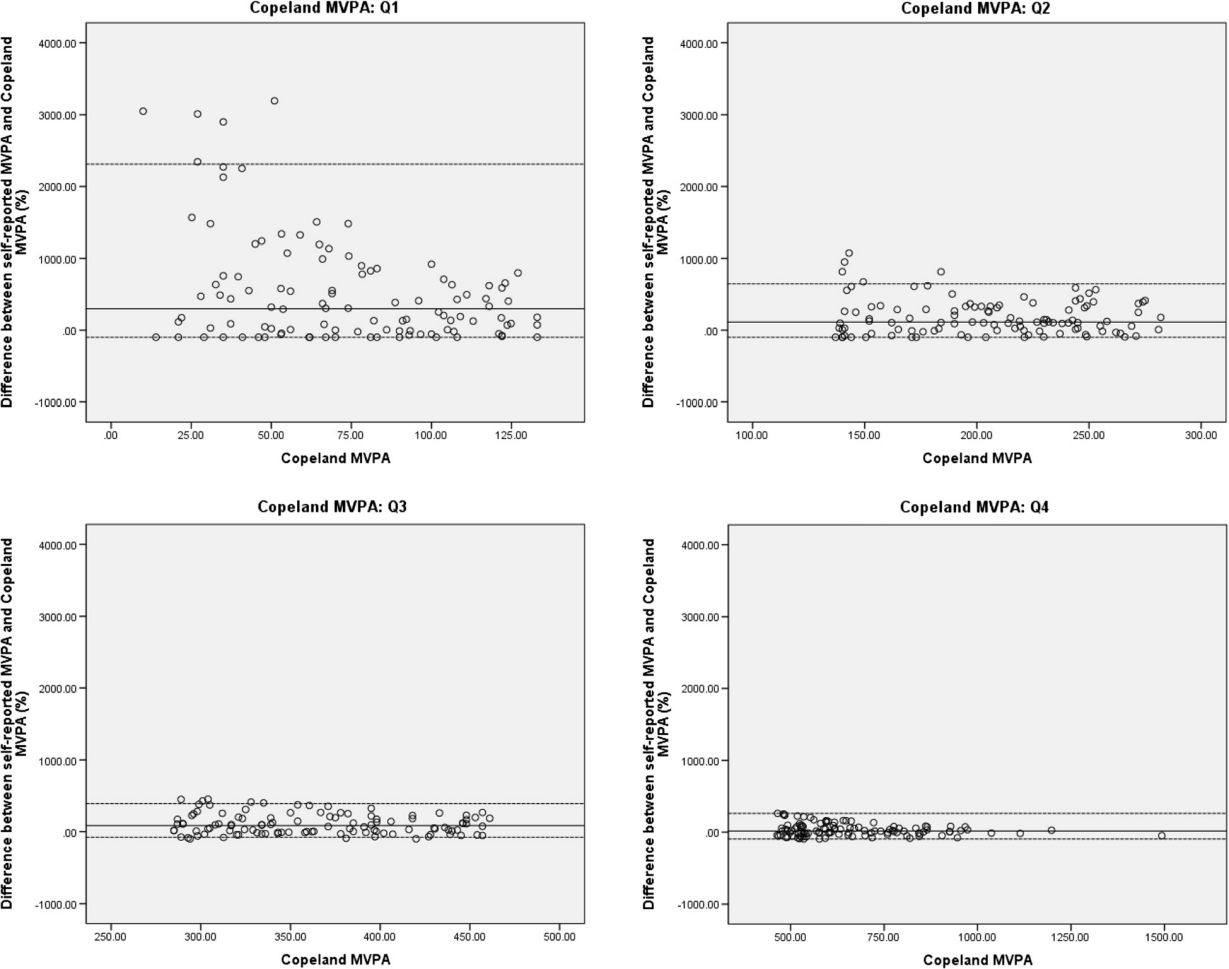
Difference between self-reported MVPA and Copeland MVPA for each quartile of Copeland MVPA. Legend: MVPA = moderate-to-vigorous physical activity; Copeland MVPA = weekly minutes of accelerometer-derived moderate-to-vigorous physical activity ≥1041 counts.min^−1^; Q1 = first quartile; Q2 = second quartile; Q3 = third quartile; Q4 = fourth quartile. y-axis represent differences between self-reported MVPA and Copeland MVPA, expressed as a percentage; x-axis represent quartiles of Copeland MVPA. Full lines represent median (M) percentage of difference, dotted lines show the 90% nonparametric limits of agreement (LOA), representing 5th and 95th percentiles (P5 and P95): **Q1**: M = 297.1; P5 = −100.0 (95% CI for P5: −100.0 - -100.0); P95 = 2311.6 (95% CI for P95: 1490.8 – 3034.4); **Q2**: M = 111.6; P5 = −100.0 (95% CI for P5: −100.0 - -95.9); P95 = 646.3 (95% CI for P95: 529.2 – 882.0); **Q3**: M = 84.5; P5 = −78.3 (95% CI for P5: −−96.0 - -49.4); P95 = 391.5 (95% CI for P95: 317.3 – 439.2); **Q4**: M = 16.4; P5 = −82.5 (95% CI for P5: −92.8 - -68.9); P95 = 219.7 (95% CI for P95: 159.3 – 249.0).

Spearman correlation coefficients between weekly minutes of self-reported and accelerometer-derived MVPA showed that for both accelerometer cut points, moderate validity of IPAQ-L was found (*ρ* = 0.36 for Freedson MVPA; *ρ* = 0.40 for Copeland MVPA). In terms of percentage of difference between self-reported MVPA and accelerometer-derived measures, the median difference was 542.9% for Freedson MVPA and 80.5% for Copeland MVPA, respectively, which shows that older adults over-reported their MVPA levels. Figures 2 and 3 illustrate the difference (expressed in terms of percentage) between weekly minutes of self-reported and accelerometer-derived MVPA for each quartile of Freedson (Figure 2) and Copeland MVPA (Figure 3), respectively. These figures depict that there is a significant decrease in over-reporting (and variability) using IPAQ-L with increasing accelerometer MVPA (in terms of percentage, lowest differences and variability in the highest quartiles).

#### Validity for measuring weekly minutes of total PA

**Figure 4:**
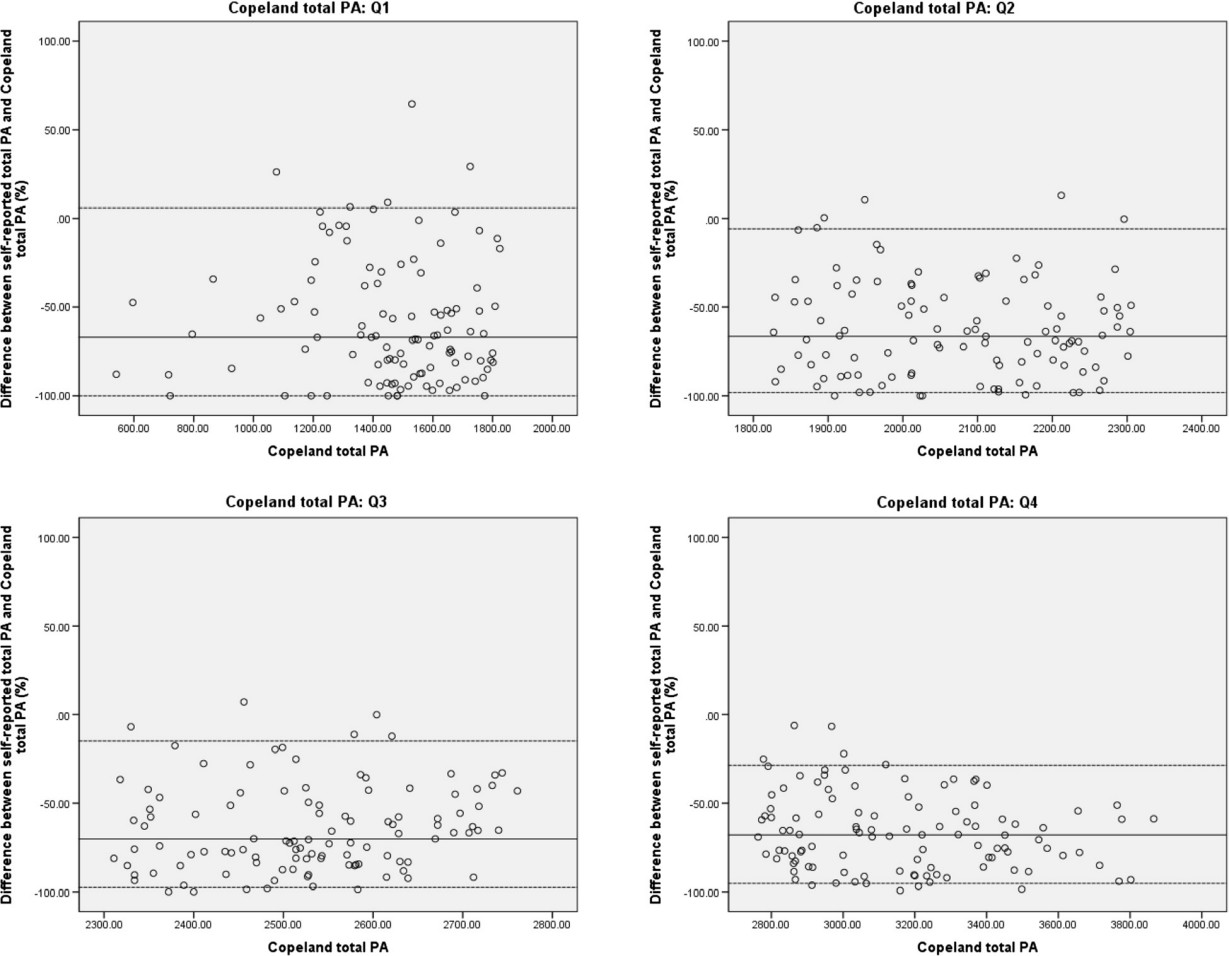
Difference between self-reported total PA and Copeland total PA for each quartile of Copeland total PA. Legend: PA = physical activity; Copeland total PA = weekly minutes of accelerometer-derived physical activity ≥50 counts.min^−1^; Q1 = first quartile; Q2 = second quartile; Q3 = third quartile; Q4 = fourth quartile. y-axis represent differences between self-reported total PA and Copeland total PA, expressed as a percentage; x-axis represent quartiles of Copeland total PA. Full lines represent median (M) percentage of difference, dotted lines show the 90% nonparametric limits of agreement (LOA), representing 5th and 95th percentiles (P5 and P95): **Q1**: M = −67.1; P5 = −100.0 (95% CI for P5: −100.0 - -96.3); P95 = 6.0 (95% CI for P95:-3.8 – 28.0); **Q2**: M = −66.6; P5 = −98.2 (95% CI for P5: −100.0 - -96.2); P95 = −5.8 (95% CI for P95: −27.9 – 5.5); **Q3**: M = −70.2; P5 = −97.5 (95% CI for P5: −99.3 – -91.8); P95 = −14.9 (95% CI for P95: −30.6 – -3.5); **Q4**: M = −67.9; P5 = −95.3 (95% CI for P5: −97.8 - -93.1); P95 = −28.6 (95% CI for P95: −35.3 - -13.7).

Spearman correlation coefficients between weekly minutes of self-reported and accelerometer-derived total PA were also similar for both cut points and also here, moderate correlations were found (*ρ* = 0.35 for Freedson total PA; *ρ* = 0.33 for Copeland total PA). In contrast with results for MVPA, older adults generally under-reported total PA using IPAQ-L. Median differences were equal for Freedson and Copeland total PA, i.e., 67.9%. This is illustrated in Figure 4 for Copeland total PA. Because plots for each quartile of Freedson total PA were very similar to these of Copeland total PA, these were not included as a separate figure, but they are shown in Additional file 3. For both Freedson and Copeland total PA, plots show a decrease in under-reporting is observed between self-reports and accelerometer-derived total PA with increasing magnitude of accelerometer-derived total PA.

### Test-retest reliability

Table 2 outlines test-retest reliability results (ICC’s) of self-reported PA, measured in the subsample (n = 29). ICC’s reflecting absolute agreement between test and retest were poor for the transportation and recreational PA domains (0.44 and 0.43, respectively), whereas work-related and domestic, as well as MVPA and total PA showed moderate-to-good test-retest reliability [39] (ICC = 0.81, 0.52, 0.63, and 0. 63, respectively). However, confidence intervals were generally wide, reflecting large variability in test-retest agreement between individuals.

**Table 2:** **Test-retest reliability of older adults’ self-reported physical activity measures**

**Self-reported PA**	**ICC**	**95% CI**
Total PA	0.633	0.358 – 0.808
MVPA	0.629	0.354 – 0.806
Work-related PA	0.812	0.640 – 0.906
Transportation PA	0.439	0.085 – 0.692
Domestic PA	0.517	0.186 – 0.741
Recreational PA	0.427	0.089 – 0.680

Results reflect Single measures Intraclass Correlation coefficients for absolute agreement.

PA, physical activity; MVPA, moderate-to-vigorous physical activity.

ICC, intra-class correlation coefficient; CI, confidence interval.

## Discussion

Worldwide, questionnaires are used to monitor health behaviors such as PA in large-scale studies. The International Physical Activity Questionnaire has been widely used in PA research and is well-accepted as a good measurement tool in adults (18-65y), but little is known on its applicability in elderly populations. To the best of our knowledge, this was the first study to examine measurement properties of an elderly-specific version of the long, last seven days version of IPAQ.

### Criterion validity

A first aim of our study was to determine criterion validity of IPAQ-L for measuring older adults’ weekly minutes of MVPA and total PA, using two different types of accelerometer cut points. A first set of cut points, defined by Freedson et al. [15], is most commonly used to define PA in adults. However, it was expected that the Freedson cut point distinguishing between light and moderate-intensity activities (1,952 counts.min^−1^) may be too high to define the threshold value for MVPA in older adults. The second accelerometer cut point set was defined by Copeland & Esliger [19] in a sample of older adults and has a lower threshold value for MVPA (1,041 counts.min^−1^).

Regarding MVPA, Spearman correlation coefficients indicated moderate-to-good validity of IPAQ-L and results were comparable for both cut points (*ρ* = 0.36 for Freedson MVPA; *ρ* = 0.40 for Copeland MVPA). As hypothesized, similar correlation coefficients for MVPA as observed in the present study were found in other validation studies using Spearman correlations between accelerometers and PA questionnaires in older adult populations (ρ = 0.43 [40]; ρ = 0.37[41]; ρ = 0.31[42]). Moreover, a review on measurement properties of PA questionnaires described that validity of questionnaires in older adult samples showed a median Spearman correlation of ρ = 0.41 [11].These moderate-to-good Spearman coefficients should be interpreted with care, since they can only suggest that IPAQ-L was relatively good at ranking individuals by their reported MVPA levels, when compared to their objectively-measured MVPA levels. In order to evaluate validity of the IPAQ-L, it is recommended that these Spearman rank order correlations are interpreted in combination with the results of the agreement plots. Plots for MVPA showed a general pattern of over-reporting of IPAQ-L and this over-reporting was higher when self-reported MVPA was compared with the Freedson MVPA, than when it was compared to the Copeland MVPA. This finding is in concordance with our hypothesis that better validity of the elderly-adapted IPAQ-L would be observed regarding Copeland MVPA, which seems a promising finding. On the other hand, it is also logical to find less over-reporting when self-reports are compared to Copeland MVPA, because the cut point has a lower threshold for defining MVPA than the Freedson MVPA cut point. However, also the Copeland MVPA cut point might not be the ideal solution for defining accelerometer MVPA in older adults. Percentages of participants in the current study reaching the Public Health Recommendations for MVPA were 27.4 % and 71.2% for Freedson and Copeland, respectively. Given that worldwide, only 30-40% of older adults reach the Public Health Recommendations for PA [43-45], the percentage found in the present study using the Copeland MVPA threshold seems rather high and Freedson MVPA levels could be a more realistic estimate of participants’ actual MVPA levels. Although it is beyond the purpose of the present study to make inferences on the applicability of cut points for defining MVPA in older adults, our findings do indicate that researchers should be cautious when selecting an appropriate accelerometer threshold for defining MVPA in older adults. Moreover, it is suggested that more research is conducted on the feasibility of different MVPA cut points in older populations.

Irrespective of the cut point applied, however, the plots for validity of IPAQ-L regarding MVPA showed that the highest over-reporting was found in older adults with lower objectively-measured activity levels (i.e., the lower quartiles shown in the plots). As PA levels show a systematic decline with increasing age [46], this may have important consequences for the utility of IPAQ-L in longitudinal studies, and in the oldest old. Some possible explanations for this systematic over-reporting can be formulated. Firstly, when participants reported to have performed a certain activity (e.g., transportation walking) on more than one day in the preceding week, the next item asked them to specify an average daily amount of time spent doing this activity. Participants may have based their response on the trip with the longest duration, whereas activities of shorter duration may have been neglected. Because self-reported minutes of MVPA were calculated by summing different types of domain-specific PA, accumulation of small over-estimations for each separate PA domain may have resulted in greater over-estimations for total self-reported MVPA. Secondly, older adults could have considered activities (particularly household chores) to be of moderate or vigorous intensity, while in reality, these should be categorized as light-intensity PA (LPA). Earlier research demonstrated that, compared to younger adults, a large proportion of older adults’ activities can be classified as LPA, while a considerably smaller amount of their time is dedicated to MVPA [47]. In contrast, IPAQ-L was mainly designed to assess MVPA and not LPA. Most likely, older adults in the present study may have wanted to report their most important daily activities and therefore also reported a considerable amount of LPA as if it was MVPA. Light-intensity PA encompasses a large part of this age group’s daily activities and is positively linked to psychosocial and physical health factors [48]. Based on this, the question arises whether or not it is more convenient to change the focus of PA questionnaires in older adults from MVPA to total PA levels, concurrently accounting for their most prevalent behavior, LPA, as well. This may also enhance content validity of IPAQ-L for estimating older adults’ total PA levels. Although our version of IPAQ-L already contains elderly-specific adaptations, specific questions on LPA (corresponding to activities with a Metabolic Equivalent (MET) value < 3) are still lacking. According to the 2011 Compendium of Physical Activities [27], items on LPA could specifically ask for home activities such as “dusting or polishing furniture” (~2.3 METs); “washing dishes, clearing dishes from the table” (~2.5 METs), or “cleaning, sweeping, light effort” (2.3 METs), as these activities are likely to be reported as moderate-intensity instead of light-intensity. Examples of non-home based light-intensity activities could include “food shopping with/without a grocery cart while standing/walking” (~2.3 METs). Hence, the inclusion of specific LPA items might minimize over-reporting bias of MVPA. In addition, if IPAQ-L would be interviewer-administered, the interviewer could preserve possible duplicate over-reporting, by prompting participants when they tend to report the same activity twice (i.e., once in the LPA response, and once in the MPA response). Further research investigating applicability of elderly-specific items on light-intensity activities is needed, however.

Regarding the present study’s validity results on total PA, Spearman correlations between self-reported and accelerometer-derived measures also indicated moderate validity of IPAQ-L (*ρ* = 0.35 for Freedson total PA; *ρ* = 0.33 for Copeland total PA). In this case, plots showed that, although variability in differences was much more modest than with MVPA results, IPAQ-L systematically underestimated actual total PA levels measured by accelerometer. Indeed, a considerable number of accelerometer activity counts were classified as LPA, whereas for self-reported activity levels, less LPA was reported. This under-reporting of specific LPA could be due to the fact that accelerometer total PA also comprises measures of the very low-intensity activities (e.g., standing upright; doing light work in the kitchen or home office; getting dressed; grooming), while IPAQ-L does not. Including items on such daily activities of very light intensity might further enhance validity of IPAQ-L towards total PA.

### Test-retest reliability

A second aim of this study was to assess test-retest reliability of IPAQ-L. ICC’s showed moderate to good test-retest reliability for self-reported work-related and domestic PA, as well as for estimates of total PA and MVPA. Concordant with our hypothesis, coefficients found in our study (i.e., ICC range 0.43-0.81) are comparable to those reported in other reliability studies on PA questionnaires for older adults. Specifically, a literature review observed median reliability ICC estimates of 0.65 in other studies conducted in older adult samples [11]. Findings of the present study showed that the highest ICC was observed for work-related PA, most likely due to low prevalence of such PA in our sample of older adults (i.e., the majority of participants reported no work-related PA on both test and retest). Poor ICC’s were observed for recreational (0.43) and transportation PA (0.44), with large inter-individual variability. This low reliability could be explained by the IPAQ-L recall reference period used in our study, i.e., the “last seven days” instead of “a usual week”. Older adults’ recreational and transportation PA may not follow a weekly returning pattern (as opposed to domestic or work-related PA, which are generally more structured). Lower test-retest reliability found for transportation and recreational PA may therefore be attributable to instability in the behaviors themselves, rather than to instability in the way of responding. For instance, given a high day-to-day variability in weather conditions in Belgium, bad weather (e.g., rain) may have discouraged older adults to engage in recreational or transportation PA during the first week of recall, while good weather may have invited them to go outdoors during the second week, or vice versa. A qualitative study in Belgian older adults supports this assumption, showing that bad weather conditions were mentioned as an influencing factor regarding engagement in transport-related walking [49]. Nonetheless, this does not mean that choosing the “last seven days” version over the “usual week” version for measuring older adults’ PA was inappropriate. In fact, older adults may experience more cognitive difficulties reporting “usual” PA levels [50], while recalling the past week was probably less cognitively challenging.

### Limitations and strengths

Some limitations of the present study should be acknowledged. A general limitation, which applies not only to this study, but to all research using accelerometers to determine older adults’ PA levels, is that cut point choice remains arbitrary and can have a substantial impact on study outcomes. Researchers investigating older adults’ PA and its correlates should be aware of this cut point issue and should cautiously select appropriate activity intensity thresholds. A first limitation that specifically applies to the present study is that we were not able to test validity of domain-specific PA, as accelerometer activity counts could not provide information on the PA context. Secondly, the reliability analyses were performed in a small sample of 29 participants, which may have reduced the power of the reliability study. Nevertheless, other studies on reliability of self-report data also reported results based on samples with fewer than 50 participants (e.g., [51-53]). Moreover, some efforts were made to make this limitation transparent to other researchers (i.e., ICC 95% confidence intervals showed that indeed, there is large inter-individual variability regarding our data). A third limitation is that using accelerometers as the objective criterion measure could have biased validity results of the present study. Specifically, accelerometers were worn at the hip and may not have appropriately captured upper-body movements, cycling and water-based activities [54], which may have consequently caused an under-estimation of time spent doing PA. However, accelerometers are able to accurately discriminate between frequency and intensity of PA and are considered to be an acceptable criterion to validate self-reported total PA and MVPA [55]. Besides, several previous studies on the validity of PA questionnaires for older adults have utilized Actigraph accelerometers as the criterion measure [13,18,40-42]. Another issue related to the accelerometer measure could be that we used weekly minutes of accelerometer MVPA to assess validity, instead of MVPA bouts lasting ≥10 consecutive minutes. This might have biased our estimate of objective MVPA and may have affected validity results. Lastly, representativeness of both validity and reliability samples should be taken into account and results should be interpreted carefully. In general, both samples were comparable with the Belgian population of older adults, but both samples had higher percentages of participants living with a partner than the general population and the reliability sample (n = 29) was higher educated. This may jeopardize generalizability to the lower-educated and those living alone. Additionally, as this study used an elderly-adapted version of IPAQ-L, our results are not generalizable to younger populations. Moreover, adaptations also included specific items on recreational cycling, which is more prevalent in Western-European countries, compared to other continents such as North America [29]. Therefore, the present study findings and utility of IPAQ-L may be less generalizable to non-Western-European populations of older adults.

Despite these limitations, using an elderly-specific measurement method to assess PA levels in Belgian older adults could also be considered a strength of the present study, because the inclusion of recreational cycling and walking pace items may have improved content validity of the questionnaire for this population. Moreover, as suggested by Cerin et al.[13], our elderly-specific version of IPAQ-L put less emphasis on items on vigorous-intensity PA by combining vigorous and moderate questions into one item. Combining these items could have reduced over-reporting bias, since it may have contributed to a minimization of cumulative over-reporting (i.e., accumulation of over-reporting on the MPA item and over-reporting on the VPA item). In addition, combining moderate- and vigorous-intensity items may have reduced the chance for duplicate reports (i.e., the same activity being reported twice: once as a response on the item regarding moderate-intensity, once on the item for vigorous-intensity). However, apart from that, researchers should be aware that despite combining moderate- and vigorous-intensity items may have been a good step towards minimization of over-reporting, still some over-reporting of MVPA may occur because older adults may have reported activities of light intensity as MVPA. A second strength of the current study is that we used an interviewer-administered version of the questionnaire. A Belgian study in adults (18-65y) observed less over-reporting bias when the interviewer-administered version versus the self-administered version of IPAQ-L was assessed [56]. Additionally, older adults may experience more cognitive difficulties when responding to a questionnaire [50], and therefore, the guidance by trained interviewers is likely to be beneficial for this older adults population, because more accurate responses can be obtained. For instance, interviewers could prompt participants to trigger their memory on activities that might have been forgotten otherwise. On the other hand, interviewers could also point out possible overlap in reports of certain activities (e.g., walking for leisure and walking for transport). Thirdly, the questionnaire used in the present study is also more relevant for use in European populations, as it assessed more complete information on European domain-specific PA through including items on recreational cycling [28,29].

## Conclusions

In summary, validity results of the present study indicated that our adapted version of the IPAQ-L should be considered with caution when estimates of older adults’ weekly minutes of MVPA are made, because participants tended to over-report their MVPA. In addition, although IPAQ-L was shown to be a valid tool to assess weekly minutes of total PA, plots showed limited agreement between weekly minutes of self-reported and accelerometer total PA. Our results suggest that for older adults, it is equally important to assess total PA next to MVPA, as a substantial part of their daily activities consists of LPA. Therefore, it would be interesting to include more items on LPA in future adapted IPAQ-L versions. Assessing older adults’ overall MVPA levels may be more appropriate by means of accelerometers, as these devices can discriminate between LPA and MVPA. Nevertheless, our adapted version of the IPAQ-L already is a good step towards higher validity of the instrument, because this version does not particularly emphasize vigorous-intensity activities, but tries to prompt older adults to report on more moderate-intensity activities. Moreover, the tool is probably acceptable for measuring older adults’ domain-specific PA and content of their daily life activities, which cannot be assessed through objective activity monitors such as the accelerometer. With regard to stability of the instrument, the present study found good reliability of IPAQ-L to assess total PA and MVPA, as well as domain-specific domestic and work-related PA. The poorer reliability scores found for older adults’ reports on transportation and recreational PA may be partly attributable to week-to-week variability in these behaviors. More research on the feasibility of IPAQ-L in populations of older adults is recommended, as the results of the present study are probably only applicable to Western-European settings and may not account for all population subgroups.

## Additional files

Additional file 1:
**Overview of older-adults-specific adaptations made to the IPAQ-L items.**
Additional file 2:
**Domain-specific content of the last 7 days IPAQ-L, adapted for Belgian older adults.**
Additional file 3:
**Difference between self-reported total PA and Freedson total PA for each quartile of Freedson total PA.** Legend: PA = physical activity; Freedson total PA = weekly minutes of accelerometer-derived physical activity ≥100 counts.min^−1^; Q1 = first quartile; Q2 = second quartile; Q3 = third quartile; Q4 = fourth quartile. y-axis represent differences between self-reported total PA and Freedson total PA, expressed as a percentage; x-axis represent quartiles of Freedson total PA. Full lines represent median (M) percentage of difference, dotted lines show the nonparametric 90% limits of agreement (LOA), representing 5th and 95th percentiles (P5 and P95): **Q1**: M = −66.7; P5 = −100.0 (95% CI for P5: −100.0 - -96.2); P95 = 7.90 (95% CI for P95:-3.8 – 28.0); **Q2**: M = −66.6; P5 = −98.2 (95% CI for P5: −100.0 - -96.2); P95 = −16.1 (95% CI for P95: −30.1 – -2.4); **Q3**: M = −70.5; P5 = −97.5 (95% CI for P5: −99.3 – -91.7); P95 = −11.7 (95% CI for P95: −27.9 – -0.2); **Q4**: M = −67.2; P5 = −95.3 (95% CI for P5: −97.8 - -92.7); P95 = −28.6 (95% CI for P95: −36.1 - -13.7).

## Abbreviations

PA: Physical activity
MVPA: Moderate-to-vigorous physical activity
IPAQ-L: International Physical Activity Questionnaire – long form
METs: Metabolic Equivalents
