# Supplementary material for: Assessment of physical activity in older Belgian adults: validity and reliability of an adapted interview version of the long International Physical Activity Questionnaire (IPAQ-L)
Source: BMC Public Health. 2015 Apr 28;15:433. doi: 10.1186/s12889-015-1785-3 (PMC4427934; doi:10.1186/s12889-015-1785-3)
Supplement: Additional file 1: — Overview of older-adults-specific adaptations made to the IPAQ-L items. [file 12889_2015_1785_MOESM1_ESM.pdf]

Supplementary File 1. Overview of older-adults-specific adaptations made to the IPAQ-L items

MODIFIED ITEM IPAQ-L FOR OLDER ADULTS (≥65y)	ORIGINAL ITEM IPAQ-L ADULTS (15-65y)
**Domain: WORK-RELATED PA**	
During the last 7 days, on how many days did you do *moderate-to-vigorous* physical activities *as part of your work*?	During the last 7 days, on how many days did you do *vigorous* physical activities *as part of your work*? / During the last 7 days, on how many days did you do *moderate* physical activities *as part of your work*?
How much time did you usually spend on one of those days doing *moderate-to-vigorous* physical activities *as part of your work*?	How much time did you usually spend on one of those days doing *vigorous* physical activities *as part of your work*? / How much time did you usually spend on one of those days doing *moderate* physical activities *as part of your work*?
If you have walked *as part of your work* during the last 7 days, how would you describe your *walking speed*? [1] high[a]; [2] moderate[b]; [3] low[c]	Not specified
**Domain: TRANSPORTATION PA**	
If you have walked *to go from place to place* during the last 7 days, how would you describe your *walking speed*? [1] high[a]; [2] moderate[b]; [3] low[c]	Not specified
**Domain: DOMESTIC PA**	
During the last 7 days, on how many days did you do *moderate-to-vigorous* physical activities *in the garden or yard*?	During the last 7 days, on how many days did you do *vigorous* physical activities *in the garden or yard*? / During the last 7 days, on how many days did you do *moderate* activities *in the garden or yard*?
How much time did you usually spend on one of those days doing *moderate-to-vigorous* physical activities *in the garden or yard*?	How much time did you usually spend on one of those days doing *vigorous* physical activities *in the garden or yard*? / How much time did you usually spend on one of those days doing *moderate* physical activities *in the garden or yard*?
**Domain: RECREATIONAL PA**	
If you have walked in your *leisure time* during the last 7 days, how would you describe your *walking speed*? [1] high[a]; [2] moderate[b]; [3] low[c]	Not specified
Not counting any bicycling you have already mentioned, during the last 7 days, on how many days did you *bicycle* for at least 10 minutes at a time *in your leisure time*?	Not specified
During the last 7 days, on how many days did you do *moderate-to-vigorous* physical activities *in your leisure time*?	During the last 7 days, on how many days did you do *vigorous* physical activities *in your leisure time*? / During the last 7 days, on how many days did you do *moderate* physical activities *in your leisure time*?
How much time did you usually spend on one of those days doing *moderate-to-vigorous* physical activities *in your leisure time*?	How much time did you usually spend on one of those days doing *vigorous* physical activities *in your leisure time*? / How much time did you usually spend on one of those days doing *moderate* physical activities *in your leisure time*?

PA=physical activity

[a]walking fast, almost running; [b]walking briskly; [c]strolling
