# Supplementary material for: Assessment of physical activity in older Belgian adults: validity and reliability of an adapted interview version of the long International Physical Activity Questionnaire (IPAQ-L)
Source: BMC Public Health. 2015 Apr 28;15:433. doi: 10.1186/s12889-015-1785-3 (PMC4427934; doi:10.1186/s12889-015-1785-3)
Supplement: Additional file 2: — Domain-specific content of the last 7 days IPAQ-L, adapted for Belgian older adults. [file 12889_2015_1785_MOESM2_ESM.pdf]

Supplementary File 2. Domain-specific content of the last 7 days IPAQ-L, adapted for Belgian older adults

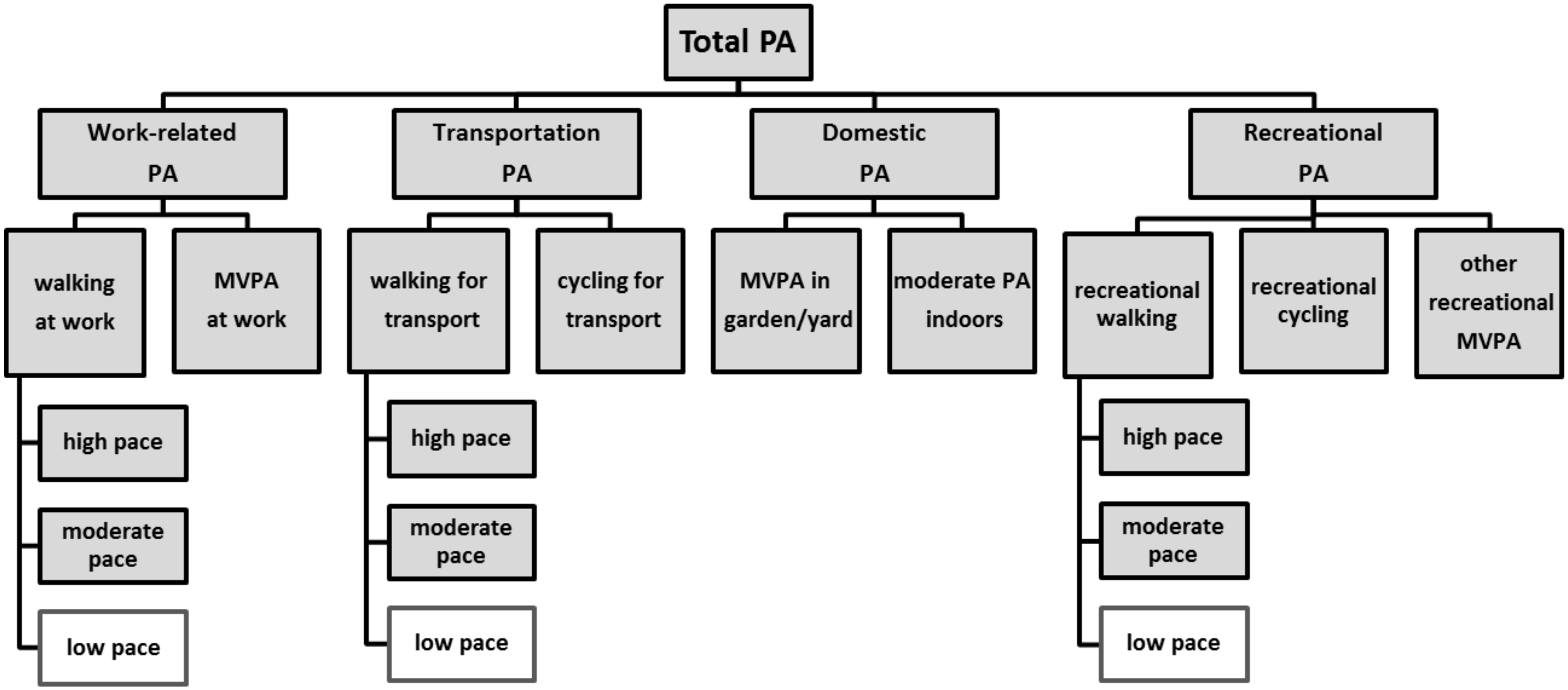

PA= physical activity; MVPA= moderate-to-vigorous physical activity

Grey boxes represent total MVPA
