# Supplementary material for: Assessment of physical activity in older Belgian adults: validity and reliability of an adapted interview version of the long International Physical Activity Questionnaire (IPAQ-L)
Source: BMC Public Health. 2015 Apr 28;15:433. doi: 10.1186/s12889-015-1785-3 (PMC4427934; doi:10.1186/s12889-015-1785-3)

Supplementary File 3. Difference between self-reported total PA and Freedson total PA (y-axis; expressed in terms of percentage) for each quartile of Freedson total PA (x-axis)

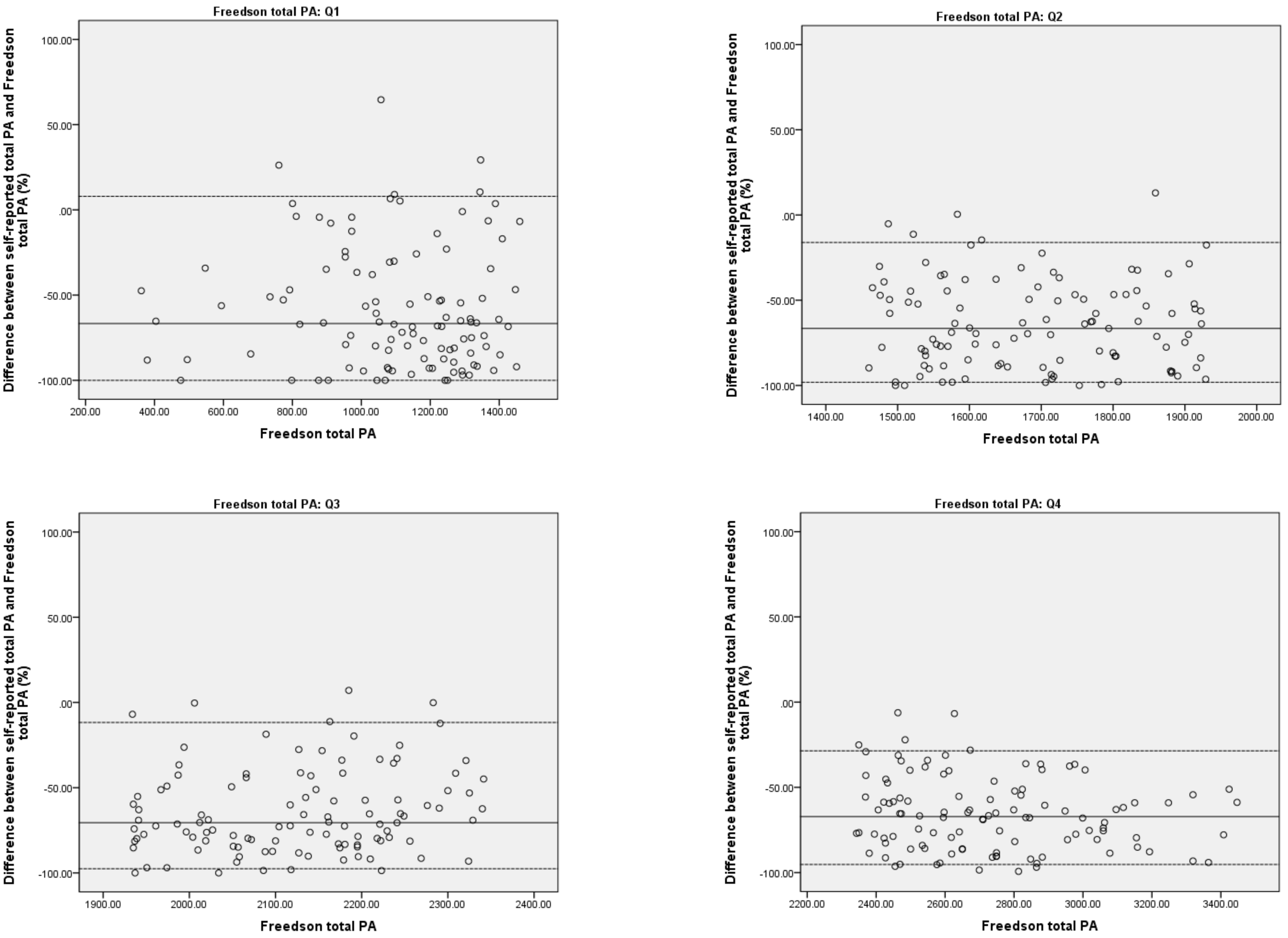

Supplement: Additional file 3: — Difference between self-reported total PA and Freedson total PA for each quartile of Freedson total PA. Legend: PA = physical activity; Freedson total PA = weekly minutes of accelerometer-derived physical activity ≥100 counts.min−1; Q1 = first quartile; Q2 = second quartile; Q3 = third quartile; Q4 = fourth quartile. y-axis represent differences between self-reported total PA and Freedson total PA, expressed as a percentage; x-axis represent quartiles of Freedson total PA. Full lines represent median (M) percentage of difference, dotted lines show the nonparametric 90% limits of agreement (LOA), representing 5th and 95th percentiles (P5 and P95): Q1: M = −66.7; P5 = −100.0 (95% CI for P5: −100.0 - -96.2); P95 = 7.90 (95% CI for P95:-3.8 – 28.0); Q2: M = −66.6; P5 = −98.2 (95% CI for P5: −100.0 - -96.2); P95 = −16.1 (95% CI for P95: −30.1 – -2.4); Q3: M = −70.5; P5 = −97.5 (95% CI for P5: −99.3 – -91.7); P95 = −11.7 (95% CI for P95: −27.9 – -0.2); Q4: M = −67.2; P5 = −95.3 (95% CI for P5: −97.8 - -92.7); P95 = −28.6 (95% CI for P95: −36.1 - -13.7). [file 12889_2015_1785_MOESM3_ESM.pdf]
